# Consensus-defined sarcopenia predicts adverse outcomes after elective abdominal surgery: meta-analysis

**DOI:** 10.1093/bjsopen/zrad065

**Published:** 2023-08-05

**Authors:** Brittany Park, Sameer Bhat, Weisi Xia, Ahmed W H Barazanchi, Christopher Frampton, Andrew G Hill, Andrew D MacCormick

**Affiliations:** Department of Surgery, Faculty of Medical and Health Sciences, The University of Auckland, Auckland, New Zealand; Department of Surgery, Faculty of Medical and Health Sciences, The University of Auckland, Auckland, New Zealand; Department of Surgery, Faculty of Medical and Health Sciences, The University of Auckland, Auckland, New Zealand; Department of Surgery, Faculty of Medical and Health Sciences, The University of Auckland, Auckland, New Zealand; Department of Surgery, Middlemore Hospital, Auckland, New Zealand; Department of Medicine, University of Otago, Christchurch, New Zealand; Department of Surgery, Faculty of Medical and Health Sciences, The University of Auckland, Auckland, New Zealand; Department of Surgery, Middlemore Hospital, Auckland, New Zealand; Department of Surgery, Faculty of Medical and Health Sciences, The University of Auckland, Auckland, New Zealand; Department of Surgery, Middlemore Hospital, Auckland, New Zealand

## Abstract

**Background:**

Sarcopenia refers to the progressive age- or pathology-associated loss of skeletal muscle. When measured radiologically as reduced muscle mass, sarcopenia has been shown to independently predict morbidity and mortality after elective abdominal surgery. However, the European Working Group on Sarcopenia in Older People (EWGSOP) recently updated their sarcopenia definition, emphasizing both low muscle ‘strength’ and ‘mass’. The aim of this systematic review and meta-analysis was to determine the prognostic impact of this updated consensus definition of sarcopenia after elective abdominal surgery.

**Methods:**

MEDLINE, Embase, Scopus, and Cochrane Central Register of Controlled Trials (CENTRAL) databases were systematically searched for studies comparing prognostic outcomes between sarcopenic *versus* non-sarcopenic adults after elective abdominal surgery from inception to 15 June 2022. The primary outcomes were postoperative morbidity and mortality. Sensitivity analyses adjusting for confounding patient factors were also performed. Methodological quality assessment of studies was performed independently by two authors using the QUality in Prognosis Studies (QUIPS) tool.

**Results:**

Twenty articles with 5421 patients (1059 sarcopenic and 4362 non-sarcopenic) were included. Sarcopenic patients were at significantly greater risk of incurring postoperative complications, despite adjusted multivariate analysis (adjusted OR 1.56, 95 per cent c.i. 1.39 to 1.76). Sarcopenic patients also had significantly higher rates of in-hospital (OR 7.62, 95 per cent c.i. 2.86 to 20.34), 30-day (OR 3.84, 95 per cent c.i. 1.27 to 11.64), and 90-day (OR 3.73, 95 per cent c.i. 1.19 to 11.70) mortality. Sarcopenia was an independent risk factor for poorer overall survival in multivariate Cox regression analysis (adjusted HR 1.28, 95 per cent c.i. 1.13 to 1.44).

**Conclusion:**

Consensus-defined sarcopenia provides important prognostic information after elective abdominal surgery and can be appropriately measured in the preoperative setting. Development of targeted exercise-based interventions that minimize sarcopenia may improve outcomes for patients who are undergoing elective abdominal surgery.

## Teaser

The consensus definition of sarcopenia was recently updated to emphasize both low muscle strength and mass. Based on this definition, sarcopenic patients had higher postoperative complication rates, higher mortality in hospital and to 3 months, and poorer overall survival after any elective abdominal surgery. Development of targeted exercised-based interventions that minimize sarcopenia may improve prognostic outcomes for patients with sarcopenia.

## Introduction

Sarcopenia refers to the generalized and often progressive loss of skeletal muscle mass that is primarily caused by ageing, but may also result from malnutrition, malignancy, or other underlying disease pathophysiology^1–3^. The European Working Group on Sarcopenia in Older People (EWGSOP) has defined sarcopenia as ‘muscle failure’ occurring secondary to muscle changes that accrue across a lifetime^4^. Sarcopenia is increasingly being recognized as a risk factor for complications after major gastrointestinal surgery^5^, over and above age, cancer diagnosis, and measures of nutritional status.

When measured as reduced skeletal muscle mass by CT, sarcopenia has recently been identified as a poor prognostic indicator in patients after major oncological and elective abdominal surgery^6,7^. The EWGSOP initially proposed that sarcopenia should be measured as reduced skeletal muscle ‘mass’ on its own due to its prognostic significance in the context of elective abdominal surgery^8^. Skeletal muscle mass is assessed using a wide range of imaging modalities, including CT, bioelectrical impedance analysis (BIA), dual-energy X-ray absorptiometry (DEXA), and ultrasonography^9^. Traditionally, cut-off values and references used to define sarcopenia have been heterogeneous, limiting its clinical utility^10^.

In 2019, however, the consensus definition was further updated to focus on reduced muscle ‘function’, measured as low muscle ‘strength’ and/or ‘performance’, together with objective radiological evidence of low muscle quantity or quality used to confirm a sarcopenia diagnosis^4^. Handgrip strength (HGS) is a universal metric with standardized cut-offs defined by the EWGSOP (based on European populations) and the Asian Working Group for Sarcopenia (AWGS) (based on Asian populations), which has been correlated with muscle ‘strength’^1,11^. In addition, gait speed (GS) has also been suggested as a measure of muscle ‘performance’ by these groups^1,11^. The addition of these standardized metrics to skeletal muscle mass may provide a more accurate method for determining the presence of sarcopenia^10^. However, the prognostic impact of this new method of measuring sarcopenia, in patients undergoing elective abdominal surgery, remains to be determined. This knowledge would aid in educating patients about their likely postoperative course, together with obtaining informed consent, and in developing targeted interventions to minimize sarcopenia for these patients^12,13^.

The hypothesis is that, compared with non-sarcopenic adults, those with sarcopenia would have lower physiological reserves and hence are likely to be at higher risk of incurring postoperative complications and mortality in both the short and long term after any elective abdominal procedure. Therefore, the aim of this systematic review and meta-analysis was to determine the prognostic impact of sarcopenia, when defined according to the EWGSOP, after elective abdominal surgery.

## Methods

The protocol for this study was registered on PROSPERO (CRD42022337609)^14^ and was reported according to the PRISMA^15^ and Meta-analysis Of Observational Studies in Epidemiology (MOOSE)^16^ guidelines (*Appendix S1*).

### Information sources

The MEDLINE (Ovid), Embase (Ovid), Scopus, and Cochrane Central Register of Controlled Trials (CENTRAL) electronic databases were systematically searched for studies published between database inception and 15 June 2022. Bibliography lists for all included studies and systematic reviews on relevant topics were also manually screened to ensure all potentially eligible studies were identified.

### Search strategy

The following keywords and Medical Subject Headings (MeSH) were combined using Boolean operators (‘AND’/’OR’), proximity search terms (‘adj3’), and the ‘explode’ function where possible: ‘physical examination’, ‘clinical marker’, ‘muscle atrophy’, ‘musc* wast*’, ‘sarcopen*', ‘laparotomy’, ‘digestive system surgical procedures’, ‘colorectal surgery’, ‘general surgery’, ‘abdom* surgery’, and ‘abdom* operation’. *Appendix S2* demonstrates the search string applied in the MEDLINE (Ovid) database. Searches were restricted to studies conducted in adult populations (greater than or equal to 18 years old) that were published in English and where the full text was accessible. There were no limitations on study design or geographical location.

### Study selection

All original studies assessing the outcomes of sarcopenic compared with non-sarcopenic adults after elective abdominal surgery were included in the present review. Sarcopenia was defined in accordance with the EWGSOP, as either reduced skeletal muscle ‘mass’ together with reduced muscle function (‘strength’ and/or ‘performance’) (definition one) or reduced skeletal muscle ‘strength’ and reduced muscle ‘quantity’ or ‘quality’ (definition two). Any imaging modality used to measure skeletal muscle quantity and quality (including CT, BIA, and ultrasonography), strength (hand dynamometer), and performance (GS test, short physical performance battery test, and the Timed ‘Up and Go’ test) was considered. In cases where studies included an identical cohort of patients originating from the same institution and across overlapping intervals of time, only the study published most recently was included.

Studies that did not use either of the EWGSOP definitions for sarcopenia were excluded, as were editorial letters, systematic and/or literature reviews, case reports or small case series (with fewer than 10 patients), studies including paediatric patients (aged less than 18 years old), and conference abstracts (where the full text could not be sourced).

### Screening process

Records were exported into EndNote X9 (Clarivate, Philadelphia, PA, USA), with duplicates being excluded via the methods of Bramer *et al*.^17^. Two reviewers screened these records independently using the Rayyan web application for systematic reviews^18^. Consensus was necessary before study inclusion, with discrepancies being resolved via senior author input as required.

### Data extraction

Study characteristics, operative details, patient selection criteria, sarcopenia definitions, patient characteristics, and postoperative morbidity and mortality outcomes were extracted (*Table S1*). Data presented as figures and/or graphs were extracted using WebPlotDigitizer (Version 4.5; Pacifica, CA, USA)^19^. Data were validated by a second author independently, with any disagreements mediated by a senior author. In cases where included studies adjusted for potentially confounding patient factors through multivariate analysis, the adjusted OR (aOR), adjusted HR (aHR), 95 per cent confidence interval (c.i.), and covariates included within each model were also extracted when reported.

### Quality assessment

Methodological quality assessment of studies was performed independently by two authors using the QUality in Prognosis Studies (QUIPS) tool^20^. Final agreement on scores was achieved through discussion, including input from a senior author if required.

### Outcome measures

The primary endpoints of this study were postoperative morbidity and mortality. Morbidity was defined according to the Clavien–Dindo (CD) classification scale^21^ and analysed as rates of overall (greater than or equal to one CD grade I–V), major (greater than or equal to one CD grade III–V), and moderate-to-minor (greater than or equal to one CD grade I–II) complications, separately. Mortality was measured during the index hospital stay (in hospital) and in the short term (at 30- and 90-day follow-up). Secondary endpoints included postoperative and total length of stay (LOS), unplanned hospital readmissions and reoperations, and long-term mortality (6 months, 1 year, and 2 years after surgery).

### Statistical analysis

All statistical analyses were performed using RStudio (Version 1.4.1106; RStudio, Boston, MA, USA)^22^.

#### Meta-analysis

Categorical and continuous data from the univariate (unadjusted) analysis in each study were reported as the frequency (*n*) and mean(s.d.) respectively. When the s.d. was not reported, it was imputed from available data using validated methods^23,24^. Continuous data reported as the median and range (or interquartile range) were converted to the mean and s.d. for the purposes of this analysis^25,26^. Continuity corrections were applied to categorical data with zero frequencies, by adding one to both the numerator and denominator^27^. Summary estimates were reported as pooled OR and mean differences (MD) for categorical and continuous endpoints respectively (with their 95 per cent c.i.). Sarcopenia prevalence and incidence rates for overall postoperative complications and in-hospital mortality were reported as the pooled prevalence and incidence (percentage) (also with their associated 95 per cent c.i.). HRs derived from Cox proportional-hazards regression models in studies reporting on overall survival were analysed where possible. Statistically significant differences were denoted by 95 per cent c.i. that did not cross the no effect line (zero for continuous endpoints and one for categorical endpoints). A random-effects meta-analysis with the DerSimonian–Laird estimator was used for all analyses to minimize the impact of the expected variability in patient demographic characteristics and sarcopenia cut-off values between studies^28^. Heterogeneity between studies for each outcome was quantified with the *I*^2^ statistic^29^, with cut-off values of 0–29, 30–49, 50–74, and 75–100 per cent being used to denote studies as being not significantly heterogeneous, moderately heterogeneous, substantially heterogeneous, and considerably heterogeneous respectively. Publication bias was assessed quantitatively using Peter’s or Egger’s regression test for categorical and continuous endpoints respectively^30,31^. *P* values of <0.050 were indicative of publication bias.

#### Subgroup and sensitivity meta-analyses

Subgroup meta-analyses were conducted based on the type of resection (gastric *versus* oesophageal *versus* colorectal *versus* liver transplantation). Sensitivity analysis of studies that performed multivariate regression analyses for any of the outcomes was also performed. The generic inverse-variance meta-analysis method was used to assign weighting to each study in this analysis^32^, with results reported as pooled aOR or aHR (with their respective 95 per cent c.i.).

## Results

### Study selection

**Fig. 1:**
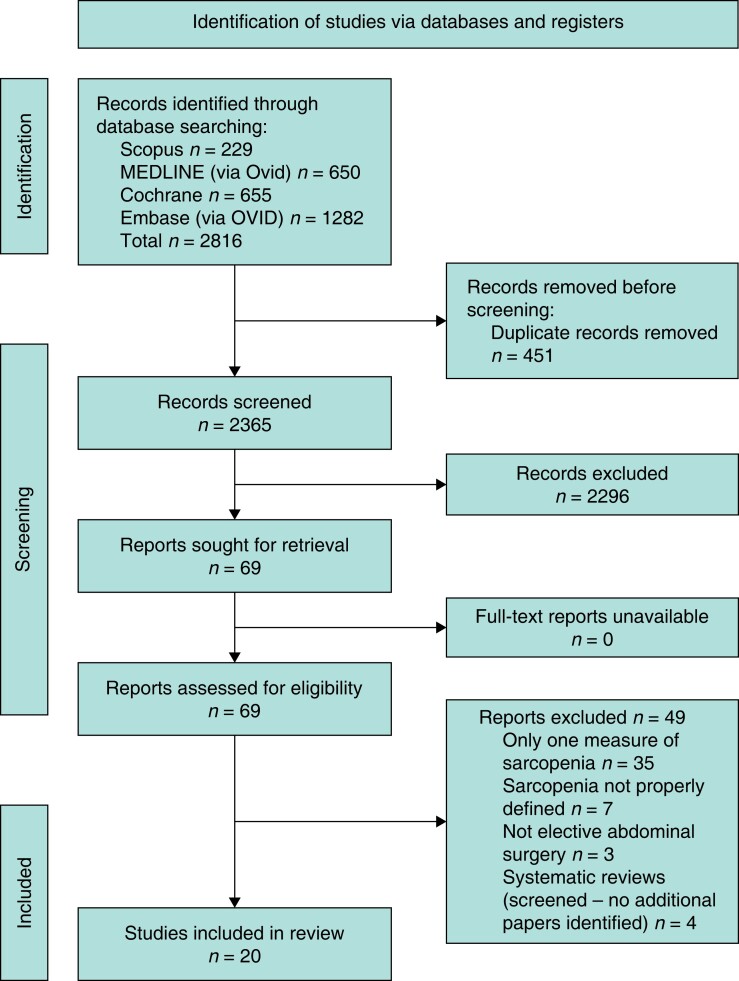
PRISMA flow diagram showing the selection process for included studies

The initial search of databases identified 2816 records, from which 24 studies were considered for inclusion^33–56^. Seven of these studies reported on identical patient cohorts and hence only the most recent of these studies, by Chen *et al*.^37^, Lou *et al*.^44^, and Makiura *et al*.^45^, were included. After exclusion of the duplicate reports^53–56^, a total of 20 studies were analysed in the present review (*Fig. 1*).

### Study characteristics

The characteristics of each study are presented in *Table 1*. Gastrectomy accounted for the majority of operations (10 studies), followed by liver transplantation (3 studies), colorectal resection and oesophagectomy (2 studies each), gynaecological operations (1 study), ‘gastrointestinal cancer surgery’ (1 study), and pancreatoduodenectomy (1 study). Elective operations were indicated for cancer in all studies aside from two^41,42^, in which liver transplantation was performed for a variety of benign and malignant indications.

**Table 1:** Characteristics of each study included in the review

Study	Country	Patient selection	Operation type	Operative indication	Study design (R/P)
Aoki 2022^33^	Japan	Inclusion: patients who underwent pancreatoduodenectomy for pancreatic head carcinoma Exclusion: not stated	Pancreatoduodenectomy	Pancreatic head carcinoma, bile duct carcinoma, intrapapillary mucinous neoplasm, ampullary carcinoma, and others (not specified)	Observational cohort study (P)
Ayçiçek 2021^34^	Turkey	Inclusion: patients with a diagnosis of ‘gastrointestinal system cancers’ Exclusion: patients with prosthesis, acute infection, severe oedema, acute cardiac diseases, pacemakers, patients who cannot cooperate, outpatient surgery, emergency surgery, palliative operations, patients who were operated on under local anaesthesia	Gastrointestinal cancer surgery	Colon cancer, gastric cancer, oesophageal cancer, rectal cancer, pancreatic cancer, other gastrointestinal cancers	Cross-sectional study (R)
Berardi 2020^35^	Italy	Inclusion: patients undergoing liver resection for malignant tumours with available preoperative CT Exclusion: patients with benign lesions, undergoing exploratory laparotomy or laparoscopy without liver resection, and extrahepatic resection, or without preoperative CT	Liver resection	Malignant tumours	Observational cohort study (P)
Chen 2016^36^	China	Inclusion: patients >18 years old, ASA grade ≤III, histologically proven gastric adenocarcinoma before surgery Exclusion: recurrence of gastric cancer, presence of motor dysfunction, cancer metastasis not curable by radical surgery	Total gastrectomy + D2 lymphadenectomy	Gastric cancer	Observational cohort study (P)
Chen 2018^37^	China	Inclusion: ≥18 years old, ASA grade ≤III, elective colorectal surgery for colorectal cancer with curative intent, had preoperative abdominal CT no more than 1 month before surgery Exclusion: palliative surgery, emergency surgery, neoadjuvant chemotherapy or radiotherapy, and aged <18 years old	Colorectal cancer surgery	Colorectal cancer	Observational cohort study (P)
Chen 2019^38^	China	Inclusion: >18 years old, ASA grade ≤III, estimated tumour size ≤5 cm, T category ≤4a, N category ≤1, where abdominal CT was available within 1 month before surgery Exclusion: palliative gastrectomy or emergency surgery, conversion to open, neoadjuvant therapy	Laparoscopic-assisted gastrectomy	Curable gastric cancer	Observational cohort study (P)
Erkul 2022^39^	Turkey	Inclusion: ≥18 years old, histologically proven gastric adenocarcinoma, scheduled for surgical treatment, and ASA grade ≤III Exclusion: emergency surgery, motor dysfunction or mobility problems, thoracotomy/thoracoscopy during surgery, had another organ malignancy	Radical gastrectomy	Gastric cancer	Observational cohort study (P)
Fukuda 2016^40^	Japan	Inclusion: patients ≥65 years old who underwent gastrectomy for gastric cancer Exclusion: patients who underwent combined resection of gastric and colorectal cancer	Gastrectomy	Gastric cancer	Observational cohort study (R)
Harimoto 2017^41^	Japan	Inclusion: patients undergoing LDLT with preoperative CT Exclusion: fulminant hepatic failure	LDLT	Meet requirements for liver transplant	Observational cohort study (P)
Kaido 2017^42^	Japan	Inclusion: aged ≥18 years old who underwent LDLT in the time interval Exclusion: patients who underwent deceased donor liver transplant and those who underwent LDLT for acute liver failure	Liver transplant	Hepatocellular diseases, carcinoma, progressive intrahepatic cholestatic diseases, alcoholic liver cirrhosis, biliary atresia, non-alcoholic steatohepatitis-associated liver cirrhosis, Budd–Chiari syndrome, polycystic liver, autoimmune hepatitis	Observational cohort study (P)
Kurita 2020^43^	Japan	Inclusion: patients with locally advanced thoracic oesophageal cancer, defined as cancer classified higher than cT2 or cN1, receiving neoadjuvant treatment and undergoing thoracoscopic-laparoscopic oesophagectomy Exclusion: not stated	Thoracoscopic-laparoscopic oesophagectomy	Oesophageal cancer	Observational cohort study (R)
Lou 2017^44^	China	Inclusion: BMI ≥23 kg/m^2^, ≥18 years old, ASA grade ≤III, proven preoperative gastric adenocarcinoma on histology Exclusion: presence of motor dysfunction, patients undergoing palliative resection	Radical gastrectomy	Gastric cancer	Observational cohort study (P)
Makiura 2018^45^	Japan	Inclusion: patients with oesophageal cancer who were scheduled to undergo oesophagectomy Exclusion: salvage surgery, dementia	Oesophageal cancer surgery	Oesophageal cancer	Observational cohort study (P)
Matsui 2021^46^	Japan	Inclusion: patients with gastric adenocarcinoma stage I to IV who underwent gastrectomy as the primary treatment Exclusion: residual gastric cancer, other cancers, preoperative paralysis, insufficient data	Gastrectomy	Primary pT2 (MP) or more advanced gastric cancer	Observational cohort study (R)
Sato 2016^47^	Japan	Inclusion: D2 or D1 gastrectomy as a primary treatment for gastric adenocarcinoma, R0 or R1 resection, ECOG 0–2, no multiple primary cancer requiring simultaneous other visceral resection, with handgrip strength and bioelectrical impedance analysis Exclusion: R2 resection, patients with double cancer, and patients in whom there were no data on skeletal muscle mass or strength	Gastrectomy	Gastric cancer	Observational cohort study (R)
Sehouli 2021^48^	Germany	Inclusion: female, >18 years old, malignant gynaecological tumour disease, who were expected to undergo elective surgery of >1 h duration Exclusion: benign tumour entity or a surgery duration <1 h	Gynaecology surgery	Gynaecological cancer	Observational cohort study (P)
Welch 2019^49^	UK	Inclusion: patients aged ≥65 years old who planned to undergo major colorectal surgery (any indication), and who were able to give informed consent Exclusion: life expectancy ≤30 days, language barrier	Colorectal surgery	‘Any colorectal indication'	Observational cohort study (P)
Zhuang 2020^50^	China	Inclusion: patients with gastric adenocarcinoma who planned to undergo elective curative surgery, were ≥18 years old, had abdominal CT within 1 month before surgery, and signed informed consent and agreed to participate in the study Exclusion: non-curative, CT performed in other institution, partial gastrectomy for remnant gastric cancer, patients with motor system diseases who were unable to complete the measurement of grip strength and gait speed	Gastrectomy	Gastric cancer	Observational cohort study (P)
Zhang 2022^52^	China	Inclusion: ≥18 years old, histologically confirmed stage I gastric adenocarcinoma, CT within 1 month before surgery, planned to receive elective curative gastric surgery Exclusion: a history of cancer, unavailable data on muscle quality and quality, unable to undergo functional assessments due to physical or mental causes	Radical gastrectomy	Stage 1 gastric cancer	Observational cohort study (P)
Zhuang 2022^51^	China	Inclusion: histological gastric adenocarcinoma, available abdominal CT, and no severe cognitive impairment Exclusion: motor system diseases, unable to complete handgrip strength or gait speed assessment, patients who received neoadjuvant chemotherapy, and patients with multiple tumours	Curative gastrectomy	Gastric cancer	Observational cohort study (P)

R, retrospective; P, prospective; LDLT, living donor liver transplantation; MP, multiple primary; ECOG, Eastern Cooperative Oncology Group.

### Quality assessment

Results of the quality assessment using the QUIPS tool are displayed in *Table S2*. Of the 20 included studies, 15 (75.0 per cent) were scored as being at low risk of bias for each of the domains. Şengül Ayçiçek *et al*.^34^ did not adjust for potentially confounding covariates in their analyses and their study population was not representative of the population of interest, resulting in a high risk of bias for each of the respective bias domains. A high risk of attrition bias was also observed in Welch *et al*.^49^ due to a large percentage of patients who declined to participate in the study by 1-week follow-up (28.6 per cent, two of seven patients).

### Sarcopenia definitions

All studies measured both skeletal muscle mass (as the skeletal muscle index (SMI)) and muscle strength (as the HGS) (*Table 2*). A total of seven studies utilized definition one for sarcopenia, whereas definition two was applied in 11 studies; the definition used was not specified in two studies^42,47^. Şengül Ayçiçek *et al*.^34^ further categorized some patients as having ‘severe’ sarcopenia, characterized by low muscle ‘strength’, ‘mass’, and ‘performance’. Skeletal muscle mass was measured with cross-sectional abdominal CT at the level of L3 in most studies (12 studies). Cut-off values for reduced muscle mass were heterogeneous across studies and were also not sex specific. Other modalities used to measure skeletal muscle mass included BIA (six studies), ultrasonography (one study), DEXA (one study), and both BIA and ultrasonography (one study). In addition, muscle quality was measured by the mean skeletal muscle attenuation (SMA; in Hounsfield units) in two studies^50–51^. The sarcopenia definition and cut-off established by Zhuang *et al*.^57^ were used in four studies. An electronic hand-held dynamometer was used to evaluate muscle strength (in kg) in most studies (17 studies), with the remainder not stating the tool that was used. Sex-specific values of less than 26 kg in men and less than 18 kg in women (11 studies)^36–38,41,42,44,45,50–53^ and less than 27 kg in men and less than 16 kg in women (4 studies)^33,34,39,43^ were the most frequently used thresholds to indicate low muscle strength. Muscle performance was estimated using either the GS (14 studies) or the Timed ‘Up and Go’ test (1 study)^48^. The cut-off for low skeletal muscle performance was 0.8 m/s across all studies in which GS was reported, consistent with definitions established by the EWGSOP and AWGS^4,11^. Most studies stated that sarcopenia assessment using the EWGSOP definition was relatively ‘easy’ to conduct and would be suitable for practical application in the elective setting.

**Table 2:** Consensus sarcopenia definitions, parameters, and cut-off values

Study	Sarcopenia definition	Definition reference	Measure	Level	Software/device	Cut-off value/definition	Formulae given/reference	Sarcopenia prevalence
Aoki 2022^33^	Low SMI + HGS	EWGSOP-1	SMI (on DEXA)	NS	NS	SMI: <7.0 kg/m^2^ (men), <6.0 kg/m^2^ (women)	SMI = appendicular lean body mass/height squared	10.6% (19/180)
			HGS	NA		<27 kg (men), <16 kg (women)	Not further specified	
			GS			≤0.8 m/s		
Ayçiçek 2021^34^	Low SMI + HGS (severe = low SMI + HGS + GS)	EWGSOP-1	SMI (on BIA, ultrasonography)	NA	Bodystat Quadscan 4000 device	SMI: <9.2 kg/m^2^ (men), <7.4 kg/m^2^ (women)	SMI (kg) = 0.566×FFMI (on BIA) Cross-sectional area of six muscle groups was determined (on ultrasonography)	28.6% (14/49)
			HGS	NA	Grip D, grip-strength dynamometer	<27 kg (men), <16 kg (women)	Measured three times, and maximum value was recorded	
			GS		NS	≤0.8 m/s (measured over 4 m)	Not further specified	
Berardi 2020^35^	Low SMI + HGS	EWGSOP-1	SMI	CT at L3	Slice-O-matic software, version 5.0 (TomoVision)	SMI: <53.5 cm^2^/m^2^ (men), <40.8 cm^2^/m^2^ (women)	Sex-specific ROC curves used	29.1% (68/234)
			HGS	NA	Dynamometer	<30 kg (men), <20 kg (women)	Two measurements taken from each hand, averaged over four readings	
			GS		NS	NS	Not further specified	
Chen 2016^36^	Low SMI + HGS and/or GS	EWGSOP-2 + AWGS	SMI	CT at L3	INFINITT PACS software, version 3.0.11.3 BN17 32 bit	SMI: <40.8 cm^2^/m^2^ (men), <34.9 cm^2^/m^2^ (women)	Height adjusted sex-specific cut-offs predetermined from ‘a large sample study from the department’	24.7% (39/158)
			HGS	NA	Electronic dynamometer	<26 kg (men), <18 kg (women)	Three trials performed in dominant hand, best result used	
			GS		NS	<0.8 m/s (measured over 6 m)	Timed 6 m walk, maximal value of three consecutive tests taken	
Chen 2018^37^	Low SMI + HGS and/or GS	EWGSOP-2 + AWGS	SMI	CT at L3	INFINITT PACS software, version 3.0.11.3 BN17 32 bit	SMI: <40.8 cm^2^/m^2^ (men), <34.9 cm^2^/m^2^ (women)	Height adjusted sex-specific cut-offs predetermined from ‘a large sample study from the department’	24.5% (92/376)
			HGS	NA	Electronic hand dynamometer	<26 kg (men), <18 kg (women)	Three trials performed in dominant hand, best result used	
			GS		NS	<0.8 m/s (measured over 6 m)	Timed 6 m walk, maximal value of three consecutive tests taken	
Chen 2019^38^	Low SMI + HGS and/or GS	EWGSOP-2 + AWGS	SMI	CT at L3	INFINITT PACS software, version 3.0.11.3 BN17 32 bit	SMI: <40.8 cm^2^/m^2^ (men), <34.9 cm^2^/m^2^ (women)	Height adjusted sex-specific cut-offs predetermined from ‘a large sample study from the department’	11.8% (37/313)
			HGS	NA	Electronic hand dynamometer	<26 kg (men), <18 kg (women)	Three trials performed in dominant hand, best result used	
			GS		NS	<0.8 m/s (measured over 6 m)	Timed 6 m walk, maximal value of three consecutive tests	
Erkul 2022^39^	Low SMI + HGS and/or GS	EWGSOP-2	SMI	CT at L3	NS	SMI: <43 cm^2^/m^2^ (men) with BMI <25 kg/m^2^ and <53 cm^2^/m^2^ with BMI ≥25 kg/m^2^, <41 cm^2^/m^2^ (women)	SMI (in cm^2^/m^2^) = cross-sectional SMA normalized for height squared; BMI- and sex-specific cut-off values used	21.2% (31/146)
			HGS	NA	Electronic hand dynamometer	<27 kg (men), <16 kg (women)	Two trials for dominant and non-dominant hands, highest measure taken	
			GS		NS	<0.8 m/s (measured over 4 m)	Not further specified	
Fukuda 2016^40^	Low SMI + HGS and/or GS	EWGSOP-1 + AWGS	SMI (on BIA)	NA	InBody 720 (Biospace)	≤8.87 kg/m^2^ (men), ≤6.42 kg/m^2^ (women)	Absolute skeletal muscle (in kg) divided by height squared	21.4% (21/98)
			HGS		Hand dynamometer	<30 kg (men), <20 kg (women)	Twice in each hand, average value across these four measurements taken	
			GS		NS	≤0.8 m/s (measured over 4 m)	Not further specified	
Harimoto 2017^41^	Low SMA + HGS	AWGS (EWGSOP-2)	SMA	CT at L3	NS	Actual SMA <75% of calculated SMA	126.9×BSA (66.2 in men), 125.6×BSA (81.1 in women)	23.5% (24/102)
			HGS	NA	Digital grip-strength dynamometer	<26 kg (men), <18 kg (women)	Measured twice, higher value used	
			GS		NS	<0.8 m/s (measured over 6 m)		
Kaido 2017^42^	Low SMM + HGS	NS	SMM (on BIA)	NA	The InBody 720	<90% of the lower limit of the standard (not further quantified)	SMM as an absolute value and as a ratio (%) compared with a standard SMM (normal range: 90–110% of the standard SMM with InBody 720)	13.9% (10/72)
			HGS		Hand dynamometer	<26 kg (men), <18 kg (women)	After confirming patients did not have evidence of hepatic encephalopathy based on clinical symptoms	
Kurita 2020^43^	Low SMI + HGS	EWGSOP-2	SMI	CT at L3	ImageJ software (NIH, MD, USA)	<47.1 cm^2^/m^2^	Optimal cut-off value determined through ROC analysis (sensitivity: 0.79; specificity: 0.52)	11.8% (19/161)
			HGS	NA	Digital handgrip dynamometer	<27 kg (men), <16 kg (women)	Twice in each hand, average value across these four measurements taken	
Lou 2017^44^	Low SMI + HGS and/or GS	EWSGOP-2 + AWGS	SMI	CT at L3	INFINITT PACS software, version 3.0.11.3 BN17 32 bit	<40.8 cm^2^/m^2^ (men), <34.9 cm^2^/m^2^ (women)	Not further specified	6.8% (14/206)
			HGS	NA	Electronic hand dynamometer	<26 kg (men), <18 kg (women)	Maximal value taken in three consecutive tests	
			GS		NS	<0.8 m/s (measured over 6 m)	Not further specified	
Makiura 2018^45^	Low SMI + HGS and/or GS	AWGS (EWGSOP-1)	SMI (on BIA)	NA	Multi-frequency bioelectrical impedance with eight electrodes (DF-860; Yamato)	<7.0 kg/m^2^ (men), <5.7 kg/m^2^ (women)	Appendicular skeletal muscle mass/height squared	31.6% (31/98)
			HGS		Grip-D grip-strength dynamometer	<26 kg (men), <18 kg (women)	Not further specified	
			GS		NS	<0.8 m/s (measured over 4 m)	Not further specified	
Matsui 2021^46^	Low SMI + HGS	EWGSOP-2	SMI	CT at L3	Ziostation software program	<42.66 cm^2^/m^2^ (men), <34.99 cm^2^/m^2^ (women)	Optimal cut-off value determined through ROC analysis	36.5% (35/96)
			HGS	NA	NS	<34.7 kg (men), <14.0 kg (women)	Measured twice in both hands, with mean value recorded	
Sato 2016^47^	Low SMI + HGS	NS	SMI (on BIA)	NA	MC-180 Body Composition Analyzer	≤17.0 kg/m^2^ (men), ≤14.0 kg/m^2^ (women)	LBM converted to LBM index (in kg/m^2^); sex-specific cut-offs	18.4% (54/293)
			HGS		Hand dynamometer	≤15.8 kg (men), ≤13.8 kg (women)	Lowest 20% of distribution of each measurement based on previously published studies	
Sehoulli 2021^48^	Low SMI	EWGSOP-1	SMI (on BIA)	NA	NS	<27%	SMM (in kg) = height squared/(BIA resistance × 0.401 + (sex × 3.825) + (age × 0.071) + 5.102) SMI = SMM/(body mass × 100)	30.1% (68/226)
			HGS	SAEHAN hand dynamometer SH5001	NS	Age-dependent low HGS (not further specified)	Age-dependent cut-offs used (measured in the dominant/stronger hand)	
			GS	NA		>9.5 s	Timed ‘Up and Go’ test (not further specified)	
Welch 2019^49^	Low BATT + HGS and/or GS	EWGSOP-1	BATT (on ultrasonography)	NA	B-mode ultrasonography (Venue 50)	<5.44 cm (men), <3.86 cm (women)	Measurements were taken in transverse plane at midpoint between greater trochanter and knee joint line Thickness of SC, RF, and VI, excluding fascia, was measured Three measurements taken on each side and mean calculated	28.6% (2/7)
			HGS		Jamar hydraulic dynamometer (Patterson Medical, Warrenville, USA)	<30 kg (men), <20 kg (women)	Participants positioned with elbows flexed to 90° with forearms in neutral position and advised to ‘squeeze as hard as they can’ Highest recording of two measurements taken on each side	
			GS		NS	<0.8 m/s (measured over 4 m)	Not further specified	
Zhuang 2020^50^	Low SMI + HGS and/or GS OR low MA + HGS	EWGSOP-2	SMI, MA	CT at L3	CT workstation (GE ADW 4.5)	SMI: <40.8 cm^2^/m^2^ (men), <34.9 cm^2^/m^2^ (women) OR MA: <38.5 HU (men), <28.6 HU (women)	Not further specified	18.9% (167/883)
			HGS	NA	Electronic hand dynamometer	<26 kg (men), <18 kg (women)	Measured on the dominant hand, best of three attempts used	
			GS		NS	≤0.8 m/s (measured over 6 m)	Not further specified	
Zhang 2022^52^	Low SMI + HGS OR low MA (muscle quality) + HGS	EWGSOP-2	SMI, MA	CT at L3	CT workstation (GE ADW 4.5)	SMI: <40.8 cm^2^/m^2^ (men), <34.9 cm^2^/m^2^ (women) OR MA: <38.5 HU (men), <28.6 HU (women)	Not further specified	14.4% (73./507)
			HGS	NA	Electronic hand dynamometer (EH101; Camry, Guangdong Province, Germany)	<26 kg (men), <18 kg (women)	Measured on the dominant hand	
Zhuang 2022^51^	Low SMI + HGS OR low MA (muscle quality) + HGS	EWGSOP-2	SMI, MA	CT at L3	CT workstation (GE ADW 4.5)	SMI: <40.8 cm^2^/m^2^ (men), <34.9 cm^2^/m^2^ (women) OR MA: <38.5 HU (men), <28.6 HU (women)	Not further specified	19.8% (241/1215)
			HGS	NA	Electronic hand dynamometer	<26 kg (men), <18 kg (women)	Maximal value of three consecutive tests in dominant hand used	

SMI, skeletal muscle index; HGS, handgrip strength; EWGSOP, European Working Group on Sarcopenia in Older People; DEXA, dual-energy X-ray absorptiometry; NS, not stated; GS, gait speed; NA, not applicable; BIA, bioelectrical impedance analysis; FFMI, fat-free muscle index; ROC, receiver operating characteristic; AWGS, Asian Working Group for Sarcopenia; SMA, skeletal muscle area; BSA, body surface area; SMM, skeletal muscle mass; NIH, National Institutes of Health; PACS, Picture Archiving and Communication System; BATT, bilateral anterior thigh thickness; SC, subcutaneous tissue; RF, rectus femoris; VI, vastus intermedius; MA, muscle attenuation; HU, Hounsfield units; LBM, lean body mass.

### Patient characteristics

**Fig. 2:**
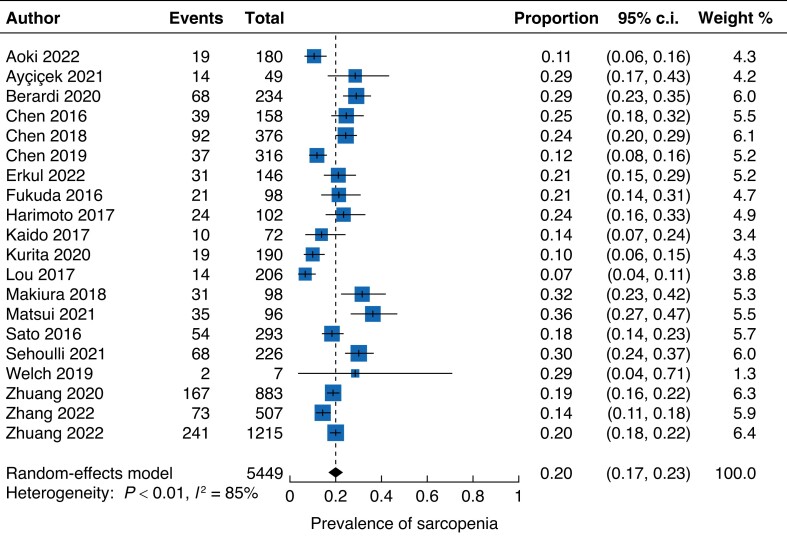
Forest plot for the pooled prevalence of sarcopenia before surgery in patients undergoing elective abdominal surgery

Elective abdominal surgery was performed in a total of 5421 patients. Patient demographic and preoperative characteristics are outlined in *Table S3*. The pooled prevalence of sarcopenia before surgery was 20 per cent (95 per cent c.i. 17 to 23 per cent; *I*^2^ = 85 per cent; *Fig. 2*). Sarcopenic patients were older (MD 6.2 years, 95 per cent c.i. 3.8 to 8.7 years; *I*^2^ = 89 per cent) and had a lower mean BMI (MD 1.8 kg/m^2^, 95 per cent c.i. 1.4 to 2.2 kg/m^2^; *I*^2^ = 56 per cent).

### Outcome measures

#### Primary endpoints

The incidence of overall morbidity after elective abdominal surgery was 29 per cent (95 per cent c.i. 20 to 44 per cent; *I*^2^ = 99 per cent; *Fig. S1a*). Sarcopenic patients were at significantly greater odds of experiencing postoperative complications compared with non-sarcopenic patients (OR 2.95, 95 per cent c.i. 2.44 to 3.57; *I*^2^ = 0 per cent; 3442 patients; *Fig. S1b*), including after adjusting for confounding patient factors (aOR 1.56, 95 per cent c.i. 1.39 to 1.76; *I*^2^ = 23 per cent; *Fig. S1c*). Higher rates of major (OR 2.46, 95 per cent c.i. 1.78 to 3.37; 2892 patients; *Fig. S1d*) and moderate-to-minor (OR 2.38, 95 per cent c.i. 1.90 to 2.99; 2666 patients; *Fig. S1e*) complications were also observed in sarcopenic compared with non-sarcopenic patients. There was very little heterogeneity detected between the studies (*I*^2^ = 6 and 0 per cent respectively). The difference in major complication rates remained despite adjusting for confounding patient factors through multivariate regression analysis in one study (aOR 4.76, 95 per cent c.i. 1.03 to 24.30)^40^.

**Fig. 3:**
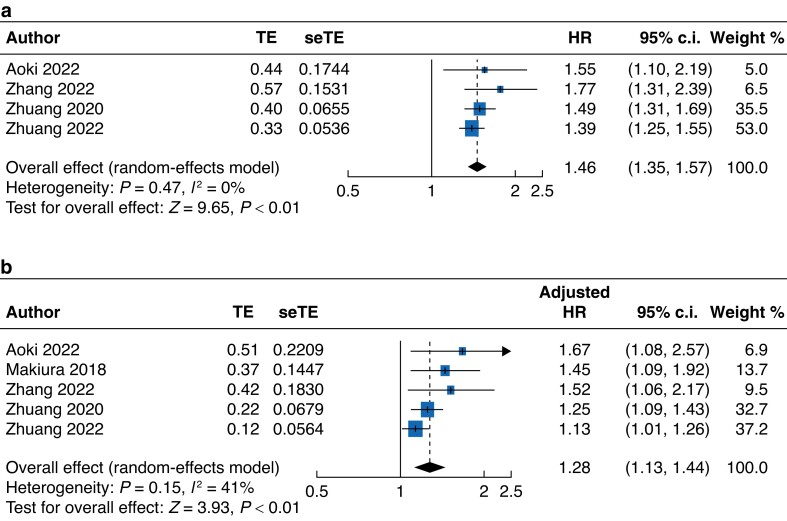
Forest plot of overall survival in sarcopenic *versus* non-sarcopenic patients **a** Univariate Cox regression analysis. **b** Multivariate Cox regression analysis. TE, mean difference; seTE, corresponding standard error.

After a median follow-up of 3.2 years, sarcopenic patients had significantly shorter overall survival after elective abdominal surgery compared with non-sarcopenic patients (HR 1.46, 95 per cent c.i. 1.35 to 1.57; *Fig. 3a*). There was no heterogeneity between the analysed studies (*I*^2^ = 0 per cent). Multivariate Cox regression analyses revealed sarcopenia as an independent risk factor for poorer overall survival (aHR 1.28, 95 per cent c.i. 1.13 to 1.44; *I*^2^ = 41 per cent; *Fig. 3b*). The incidence of in-hospital mortality after elective abdominal surgery in the overall cohort was 1 per cent (95 per cent c.i. 0 to 6 per cent; *I*^2^ = 88 per cent; *Fig. S2a*), with sarcopenic patients being at significantly greater risk of in-hospital mortality (OR 7.62, 95 per cent c.i. 2.86 to 20.34; 1711 patients; *Fig. S2b*). This difference in mortality risk persisted at both the 30-day (OR 3.84, 95 per cent c.i. 1.27 to 11.64; 415 patients; *Fig. S2c*) and 90-day (OR 3.73, 95 per cent c.i. 1.19 to 11.70; 336 patients; *Fig. S2d*) follow-up. No heterogeneity was detected between studies at any follow-up interval (*I*^2^ = 0 per cent).

#### Secondary endpoints

Results of the meta-analyses for secondary endpoints are displayed in *Table 3*. Sarcopenic patients had higher unplanned hospital readmission rates (OR 2.23, 95 per cent c.i. 1.38 to 3.60; *I*^2^ = 7 per cent) and postoperative (MD 2.94, 95 per cent c.i. 1.08 to 4.80 days; *I*^2^ = 62 per cent) and total (MD 2.66, 95 per cent c.i. 0.58 to 4.75 days; *I*^2^ = 62 per cent) hospital LOS compared with non-sarcopenic patients. Long-term mortality risk at 6 months (OR 4.00, 95 per cent c.i. 1.15 to 13.88)^39^ and 1 year (OR 4.77, 95 per cent c.i. 1.60 to 14.17)^48^ after surgery was higher in sarcopenic compared with non-sarcopenic patients, although these outcomes were reported in only one study each. Significantly higher unplanned readmissions at 90-day follow-up were also demonstrated among sarcopenic patients in multivariate regression analysis in one study (aOR 3.71, 95 per cent c.i. 1.29 to 11.05)^45^.

**Table 3:** Meta-analyses of secondary endpoints in sarcopenic *versus* non-sarcopenic patients after elective abdominal surgery

Outcome	No. of studies	Reference(s)	Effect size (95% c.i.)*	*I* ^2^, %
Unplanned hospital readmissions†‡	7	^35,37–39,44,45^§^,52^	2.23 (1.38,3.60)¶	7
Unplanned reoperations‡	3	^34,38,39^	1.66 (0.42,6.51)	0
Length of stay (days)#				
Postoperative	7	^38–41,44,45,52^	2.94 (1.08,4.80)¶	62
Total	3	^34,35,37^	2.66 (0.58,4.75)¶	62
Postoperative mortality (long term)‡				
6 months	1	^41^	4.00 (1.15,13.88)¶	–
1 year	1	^52^	4.77 (1.60,14.17)¶	–
2 years	1	^45^	2.18 (0.92,5.17)	–

*An OR >1 and mean difference >0 favour sarcopenic patients, whereas OR <1 and mean difference <0 favour non-sarcopenic patients. †Within 30 days of hospital discharge (unless otherwise stated). ‡Effect size presented as the OR. §Within 90 days of hospital discharge. ¶95 per cent c.i. that did not cross the no effect line (0 for continuous endpoints and 1 for categorical endpoints) and thus considered to indicate a statistically significant difference. #Effect size presented as the mean difference. *I*^2^ is the test for statistical heterogeneity between studies.

### Publication bias

Publication bias was not evident for overall morbidity (*P* = 0.15) and major complications (*P* = 0.34). The remainder of primary and secondary endpoints were infrequently reported and hence publication bias could not be determined for these outcomes.

### Subgroup meta-analyses

Pancreatoduodenectomy and gynaecological procedures were analysed in one study each and thus separate subgroup meta-analyses could not be performed for these operations due to the insufficient number of studies.

#### Gastric

Congruent with the overall analysis, sarcopenic patients undergoing gastric surgery were significantly more likely to incur any postoperative morbidity (OR 2.99, 95 per cent c.i. 2.34 to 3.83; *I*^2^ = 0 per cent; *Table S4*), as well as major (OR 2.66, 95 per cent c.i. 1.77 to 4.01; *I*^2^ = 0 per cent) and moderate-to-minor (OR 2.53, 95 per cent c.i. 1.94 to 3.29; *I*^2^ = 8 per cent) complications. Higher rates of in-hospital (OR 5.86, 95 per cent c.i. 1.77 to 19.37; *I*^2^ = 0 per cent) and 1-year (OR 4.77, 95 per cent c.i. 1.60 to 14.17) mortality and longer postoperative LOS (MD 2.05 days) were also observed in the sarcopenic cohort.

#### Oesophageal

Higher rates of overall morbidity (OR 2.75, 95 per cent c.i. 1.12 to 6.73) and unplanned readmissions (OR 2.96, 95 per cent c.i. 1.22 to 7.22; *I*^2^ = 0 per cent) were demonstrated among sarcopenic patients after oesophageal surgery (*Table S5*). There were no differences for other endpoints.

#### Colorectal

Chen *et al.*^37^ showed that sarcopenic patients have higher rates of overall postoperative morbidity (OR 2.22, 95 per cent c.i. 1.35 to 3.66) after colorectal resections. Rates of unplanned readmissions and total hospital LOS did not differ between sarcopenic and non-sarcopenic cohorts (*Table S6*).

#### Liver transplantation

Outcomes among sarcopenic patients undergoing liver transplantation were similar to those of the overall analysis (*Table S7*). This included higher rates of overall postoperative morbidity (OR 4.00, 95 per cent c.i. 2.40 to 6.66), major (OR 5.71, 95 per cent c.i. 2.05 to 15.94) and moderate-to-minor (OR 2.23, 95 per cent c.i. 1.18 to 4.21) morbidity, and in-hospital mortality (OR 13.11, 95 per cent c.i. 2.36 to 72.96). Higher rates of 30-day (OR 3.38, 95 per cent c.i. 1.01 to 11.30), 90-day (OR 3.73, 95 per cent c.i. 1.19 to 11.70), and 6-month (OR 4.00, 95 per cent c.i. 1.15 to 13.88) mortality were also demonstrated among sarcopenic patients.

### Sensitivity analysis

Multivariate regression analyses were performed in all but three studies^36,44,51^. These studies adjusted for a range of potentially confounding preoperative and patient demographic factors (refer to *Table S8* for the list of included covariates).

## Discussion

This systematic review and meta-analysis has shown that sarcopenia, when defined using low muscle mass and function (strength and/or performance) according to the EWGSOP, provides important prognostic information after elective abdominal surgery. Sarcopenic patients were at consistently greater risk of incurring postoperative complications, regardless of type of elective operation and despite adjustment for confounding preoperative and patient factors. Furthermore, postoperative mortality rates were consistently higher among sarcopenic patients up to 1-year follow-up, with sarcopenia identified as an independent risk factor for poorer overall survival after elective abdominal surgery.

Current consensus statements do not recommend the use of muscle mass on its own to define sarcopenia because of a non-linear relationship between muscle mass and function^4,10,11^. Muscle mass is measured using different modalities, such as axial CT, BIA, ultrasonography, and/or DEXA, some of which require specialized training and thus may be more costly and less straightforward to interpret. Furthermore, the lack of standardized cut-off values for the SMI, which is the most frequently used metric to quantify muscle mass, has hindered its clinical utility^4^. Standardization of measures and cut-offs for skeletal muscle mass may be the key in achieving translation of the revised EWGSOP definitions for sarcopenia into routine clinical practice. The updated EWGSOP guidelines place an increased emphasis on muscle ‘strength’ in sarcopenia diagnosis, with recognition that strength may be better than mass at predicting adverse outcomes^4^. Consistent with the updated proposal made by the EWGSOP, the combination of these measures may provide the most accurate assessment of sarcopenia^10^.

Several studies in the present review have highlighted that sarcopenia, when defined according to the EWGSOP, is relatively simple to assess in the clinic setting before an elective abdominal operation^35–37,41,43,47,50^. Measures of muscle strength and performance, such as HGS and GS, are inexpensive and may be determined non-invasively using an electronic hand-held dynamometer and simple timed gait test respectively. Additionally, the development of standardized cut-off values for HGS and GS by consensus groups, such as the EWGSOP and AWGS, have facilitated the interpretation of muscle strength and performance^4,11^.

Given the high preoperative prevalence of sarcopenia of 20 per cent among patients undergoing elective abdominal surgery, early recognition and treatment of sarcopenia may allow for optimization of postoperative outcomes^12^. Simple exercise interventions have consistently been shown to result in significant improvements to muscle strength, muscle mass, and muscle performance, such as in the timing of sit-to-stand and walking speed tests in a recent review^13^. Compared with preoperative/prehabilitation interventions, those commenced in the early postoperative interval (within 6 weeks after surgery) led to greater improvements in muscle mass and GS, whilst late postoperative interventions (more than 6 weeks after surgery) were more effective at reducing timed gait test scores^13^. Structured exercise-based programmes are increasingly being advocated for as part of enhanced recovery protocols (ERPs) for patients undergoing major non-cardiac surgery^58^. In a randomized trial, Northgraves *et al*.^59^ demonstrated a trend toward improvements in GS and other timed tests in patients who have trialled prehabilitation exercises before undergoing elective colorectal surgery. However, 42 per cent of patients were excluded as they could not participate in such interventions due to extremely short elective surgery wait times (of less than 2 weeks). It is also anticipated that patient compliance may be higher with the introduction of postoperative rather than preoperative/prehabilitation exercise-based regimens in the context of elective surgery, given the interval of postoperative monitoring required after surgery. Furthermore, in cachectic patients with cancer, such prehabilitation exercise-based interventions may be counteracted by the cancer pathophysiology itself, which suggests that postoperative interventions may show greater efficacy in improving sarcopenia for these patients. Therefore, the utility of postoperative exercise interventions when incorporated into ERPs, particularly in the interval immediately after surgery, needs to be further assessed for their potential to improve outcomes for sarcopenic patients after elective abdominal surgery.

Elective abdominal surgery was indicated for cancer in all but two studies^41,42^. Cancer patients may have reduced physiological reserves secondary to their underlying disease pathophysiology, such as cancer-related cachexia and malnutrition^3^. This may result in a diminished ability to respond to the stress response initiated by the ‘surgical insult’, making these patients more vulnerable to adverse perioperative and postoperative outcomes^3^. In turn, major abdominal surgery is known to cause persistent inflammation and immunosuppression through the release of pro-inflammatory cytokines, which results in risk of prolonged critical illness and mortality^60^. Such risk is compounded in sarcopenic patients who already have decreased physiological and homeostatic reserves. Failure to recognize the higher needs of these sarcopenic patients may translate into inadequate patient-centred planning, increased hospital costs, and incidence of ‘failure to rescue’, which refers to the inability of the health service to prevent death after the development of a postoperative complication^61^. Early diagnosis of sarcopenia ensures that patient-centred bundles of care, comprising supplemental nutrition, exercise-based interventions, shared care with geriatric and medical services, and early recognition and management of postoperative complications, are instituted at an earlier stage to optimize outcomes for sarcopenic patients with cancer after elective abdominal surgery^61^.

Included studies were most commonly conducted in Eastern Asia (eight studies in Japan and seven studies in China). Whilst this may have contributed to the low heterogeneity observed between studies, the prevalence of sarcopenia is known to vary based on ethnicity and race^62^, signifying that the results may not be generalizable to patient populations outside of these geographical regions. In addition, cut-off values for the sarcopenic parameters of SMI, HGS, and GS were originally defined in European populations by the EWGSOP and thus may be less applicable to patients who are from varying ethnic and/or racial backgrounds. Therefore, further work is required to establish optimal cut-off thresholds for sarcopenic parameters and whether sarcopenia may have equivalent prognostic implications after elective abdominal surgery in ethnically and racially diverse patient populations.

This review has several limitations. There was a wide degree of variability in modalities and parameters used to quantify muscle mass, strength, and performance between studies, as well as in the cut-off values used to define sarcopenia. In addition, elective abdominal operations consisted of a range of different procedures, likely with differing risk profiles. Nonetheless, a random-effects model was used for all meta-analyses, together with subgroup analyses stratified by the type of resection (gastric *versus* oesophageal *versus* colorectal *versus* liver transplantation). Results of the subgroup analyses did not differ from the overall analysis, suggesting that variations in the type of operation performed are unlikely to have contributed to the observed heterogeneity. Despite this, heterogeneity between the studies was low and not considered statistically significant for most endpoints. Lastly, several studies reported on identical patient cohorts and, to minimize multiple publication bias, only those without overlapping patient populations could be included in the meta-analysis, limiting the number of available studies for comparison.

The revised consensus definition of sarcopenia by the EWGSOP provides important prognostic information after elective abdominal surgery. According to this definition, sarcopenic patients were at significantly greater risk of incurring any postoperative complication, regardless of the type of elective abdominal procedure performed and despite adjusting for confounding preoperative and patient factors. Sarcopenia was also identified as an independent risk factor for poorer overall survival after elective abdominal surgery. Consensus-defined sarcopenia can be appropriately measured in the preoperative setting and early identification of sarcopenia may aid in the development of targeted exercise-based interventions aimed at minimizing sarcopenia^12,13^, which could improve outcomes for patients undergoing elective abdominal surgery.

## Supplementary Material

zrad065_Supplementary_Data

## Data Availability

All data used in this manuscript are available from the corresponding author on reasonable request.
