# Supplementary material for: Consensus-defined sarcopenia predicts adverse outcomes after elective abdominal surgery: meta-analysis
Source: BJS Open. 2023 Aug 5;7(4):zrad065. doi: 10.1093/bjsopen/zrad065 (PMC10404004; doi:10.1093/bjsopen/zrad065)
Supplement: zrad065_Supplementary_Data [file zrad065_supplementary_data.docx]

**Title**

Consensus-defined sarcopenia predicts adverse outcomes after elective abdominal surgery: meta-analysis

**Authors**

Brittany Park MBChB^1^, Sameer Bhat MBChB BMedSc(Hons)^1^, Weisi Xia MBChB PhD^1^, Ahmed W.H. Barazanchi MBChB FRACS^1,2^, Christopher Frampton PhD^3*^, Andrew G. Hill MBChB MD FRACS FACS^1,2^, Andrew D. MacCormick MBChB PhD FRACS^1,2^

^1^ Department of Surgery, Faculty of Medical and Health Sciences, The University of Auckland, Waipapa Taumata Rau, Auckland, Aotearoa New Zealand

^2^ Department of Surgery, Middlemore Hospital, Te Whatu Ora Counties Manukau, Auckland, Aotearoa New Zealand

^3^ Department of Medicine, University of Otago, Christchurch, Aotearoa New Zealand

**Corresponding author:** Dr Brittany Park, 85 Grafton Road, Grafton, Auckland 1023, New Zealand

**Supplementary Materials - Index**

| **Supplementary Appendixes** |  |
| --- | --- |
| Appendix S1 | *1* |
| Appendix S2 | *7* |
| **Supplementary Figures and Tables** |  |
| Table S1 | *8* |
| Table S2 | *9* |
| Table S3 | *10* |
| Figure S1 | *12* |
| Figure S2 | *13* |
| Table S4 | *14* |
| Table S5 | *16* |
| Table S6 | *18* |
| Table S7 | *20* |
| Table S8 | *22* |

**Supplementary Appendixes**

**Appendix S1.** Preferred Reporting Items for Systematic Reviews and Meta-Analyses (PRISMA) and Meta-analysis of Observational Studies in Epidemiology (MOOSE) checklists.

| **Section/topic** | **#** | **Checklist item** | **Reported on page #** |
| --- | --- | --- | --- |
| **TITLE** | | |  |
| Title | 1 | Identify the report as a systematic review, meta-analysis, or both. | 1 (Title Page) |
| **ABSTRACT** | | |  |
| Structured summary | 2 | Provide a structured summary including, as applicable: background; objectives; data sources; study eligibility criteria, participants, and interventions; study appraisal and synthesis methods; results; limitations; conclusions and implications of key findings; systematic review registration number. | 2 (Abstract page) |
| **INTRODUCTION** | | |  |
| Rationale | 3 | Describe the rationale for the review in the context of what is already known. | 4 |
| Objectives | 4 | Provide an explicit statement of questions being addressed with reference to participants, interventions, comparisons, outcomes, and study design (PICOS). | 5 |
| **METHODS** | | |  |
| Protocol and registration | 5 | Indicate if a review protocol exists, if and where it can be accessed (e.g., Web address), and, if available, provide registration information including registration number. | 6 |
| Eligibility criteria | 6 | Specify study characteristics (e.g., PICOS, length of follow-up) and report characteristics (e.g., years considered, language, publication status) used as criteria for eligibility, giving rationale. | 6-7 |
| Information sources | 7 | Describe all information sources (e.g., databases with dates of coverage, contact with study authors to identify additional studies) in the search and date last searched. | 6 |
| Search | 8 | Present full electronic search strategy for at least one database, including any limits used, such that it could be repeated. | 6, Appendix S2 |
| Study selection | 9 | State the process for selecting studies (i.e., screening, eligibility, included in systematic review, and, if applicable, included in the meta-analysis). | 6-7 |
| Data collection process | 10 | Describe method of data extraction from reports (e.g., piloted forms, independently, in duplicate) and any processes for obtaining and confirming data from investigators. | 7-8 |
| Data items | 11 | List and define all variables for which data were sought (e.g., PICOS, funding sources) and any assumptions and simplifications made. | 8, Supplementary Table 1 |
| Risk of bias in individual studies | 12 | Describe methods used for assessing risk of bias of individual studies (including specification of whether this was done at the study or outcome level), and how this information is to be used in any data synthesis. | 8 |
| Summary measures | 13 | State the principal summary measures (e.g., risk ratio, difference in means). | 8-10 |
| Synthesis of results | 14 | Describe the methods of handling data and combining results of studies, if done, including measures of consistency (e.g., I^2^) for each meta-analysis. | 9-10 |
| **Section/topic** | **#** | **Checklist item** | **Reported on page #** |
| Risk of bias across studies | 15 | Specify any assessment of risk of bias that may affect the cumulative evidence (e.g., publication bias, selective reporting within studies). | 9 |
| Additional analyses | 16 | Describe methods of additional analyses (e.g., sensitivity or subgroup analyses, meta-regression), if done, indicating which were pre-specified. | 10 |
| **RESULTS** | | |  |
| Study selection | 17 | Give numbers of studies screened, assessed for eligibility, and included in the review, with reasons for exclusions at each stage, ideally with a flow diagram. | 11, Figure 1 |
| Study characteristics | 18 | For each study, present characteristics for which data were extracted (e.g., study size, PICOS, follow-up period) and provide the citations. | 11, Table 1 |
| Risk of bias within studies | 19 | Present data on risk of bias of each study and, if available, any outcome level assessment (see item 12). | 11, Supplementary Table 2 |
| Results of individual studies | 20 | For all outcomes considered (benefits or harms), present, for each study: (a) simple summary data for each intervention group (b) effect estimates and confidence intervals, ideally with a forest plot. | 12-15, Tables 2 & 3, Supplementary Table 3, Figures 2-3, Supplementary Figures 1-2, Table 3 |
| Synthesis of results | 21 | Present results of each meta-analysis done, including confidence intervals and measures of consistency. | 12-15, Tables 2-3, Figures 2-3, Supplementary Table 3, Supplementary Figures 1-2 |
| Risk of bias across studies | 22 | Present results of any assessment of risk of bias across studies (see Item 15). | 15 |
| Additional analysis | 23 | Give results of additional analyses, if done (e.g., sensitivity or subgroup analyses, meta-regression [see Item 16]). | 15-16, Supplementary Tables 4-8 |
| **DISCUSSION** | | |  |
| Summary of evidence | 24 | Summarize the main findings including the strength of evidence for each main outcome; consider their relevance to key groups (e.g., healthcare providers, users, and policy makers). | 17-21 |
| Limitations | 25 | Discuss limitations at study and outcome level (e.g., risk of bias), and at review-level (e.g., incomplete retrieval of identified research, reporting bias). | 20-21 |
| Conclusions | 26 | Provide a general interpretation of the results in the context of other evidence, and implications for future research. | 22 |
| **FUNDING** | | |  |
| Funding | 27 | Describe sources of funding for the systematic review and other support (e.g., supply of data); role of funders for the systematic review. | 23 |

| **Item No** | **Recommendation** | **Reported on Page No** |
| --- | --- | --- |
| Reporting of background should include | | |
| 1 | Problem definition | 4-5 |
| 2 | Hypothesis statement | 5 |
| 3 | Description of study outcome(s) | 8 |
| 4 | Type of exposure or intervention used | 6-7 |
| 5 | Type of study designs used | 6 |
| 6 | Study population | 6-7 |
| Reporting of search strategy should include | | |
| 7 | Qualifications of searchers (eg, librarians and investigators) | 1 (Title page) |
| 8 | Search strategy, including time period included in the synthesis and key words | 6, Appendix S2 |
| 9 | Effort to include all available studies, including contact with authors | 6-7 |
| 10 | Databases and registries searched | 6 |
| 11 | Search software used, name and version, including special features used (eg, explosion) | - |
| 12 | Use of hand searching (eg, reference lists of obtained articles) | 6, Figure 1 |
| 13 | List of citations located and those excluded, including justification | 11, Figure 1 |
| 14 | Method of addressing articles published in languages other than English | 6-7 |
| 15 | Method of handling abstracts and unpublished studies | 6-7 |
| 16 | Description of any contact with authors | - |
| Reporting of methods should include | | |
| 17 | Description of relevance or appropriateness of studies assembled for assessing the hypothesis to be tested | 11, Table 1 |
| 18 | Rationale for the selection and coding of data (eg, sound clinical principles or convenience) | - |
| 19 | Documentation of how data were classified and coded (eg, multiple raters, blinding and interrater reliability) | - |
| 20 | Assessment of confounding (eg, comparability of cases and controls in studies where appropriate) | 9-10, 16, Supplementary Table 8 |
| 21 | Assessment of study quality, including blinding of quality assessors, stratification or regression on possible predictors of study results | 8, 11, Supplementary Table 2 |
| 22 | Assessment of heterogeneity | 13-16, Supplementary Figures 1-2, Figures 2-3, Supplementary Tables 3-7 |
| 23 | Description of statistical methods (eg, complete description of fixed or random effects models, justification of whether the chosen models account for predictors of study results, dose-response models, or cumulative meta-analysis) in sufficient detail to be replicated | 8-10 |
| 24 | Provision of appropriate tables and graphics | Tables 1-3, Figures 2-3, Supplementary Tables 3-8 |
| Reporting of results should include | | |
| 25 | Graphic summarizing individual study estimates and overall estimate | Figures 2-3, Supplementary Figures 1-2 |
| 26 | Table giving descriptive information for each study included | Table 1 |
| 27 | Results of sensitivity testing (e.g, subgroup analysis) | 15-16, Supplementary Tables 4-8 |
| 28 | Indication of statistical uncertainty of findings | 13-16, Supplementary Tables 4-7, Figures 2-3, Table 3 |

From: Stroup DF, Berlin JA, Morton SC, et al, for the Meta-analysis Of Observational Studies in Epidemiology (MOOSE) Group. Meta-analysis of Observational Studies in Epidemiology. A Proposal for Reporting. JAMA. 2000;283(15):2008-2012. doi: 10.1001/jama.283.15.2008.

# **Appendix S2.** Search string exemplar applied to the MEDLINE (OVID) electronic database

The search was conducted on June 26, 2022.

**Ovid MEDLINE(R) Epub Ahead of Print, In Process & Other Non-Indexed Citations, Ovid MEDLINE (R) Daily, and Ovid MEDLINE (R) 1946-Present:**

1 exp physical examination

2 (clinical OR functional OR physical) adj3 (marker* OR measure* OR predictor* OR test* OR exam* OR analysis OR parameter*)).mp

3 1 OR 2

4 muscle atrophy/ OR exp sarcopenia

5 (sarcopaeni* OR sarcopeni* OR (musc* adj3 (wast* OR atrophy* OR mass OR size OR function* OR quality OR composition))).mp

6 4 OR 5

7 exp laparotomy

8 exp Digestive system surgical procedures/ OR exp Urologic surgical procedures/ OR gynaecologic surgical procedures

9 exp colorectal surgery/ OR exp general surgery/ OR exp gynecology/ OR exp urology/ OR exp emergency laparotomy/ OR exp acute laparotomy

10 (abdom* surgery OR abdom* operation OR laparotomy).mp

11 ((gastrointestinal OR GI OR urolog* OR gynaecolog* OR colorectal OR digestive system(and surg*)).mp

12 7 OR 8 OR 9 OR 10 OR 11

13 3 AND 6 AND 12

14 limit 13 to (English language and adults)

mp=title, abstract, original title, name of substance word, subject heading word, floating sub-heading word, keyword heading word, organism supplementary concept word, protocol supplementary concept word, rare disease supplementary concept word, unique identifier, synonyms.

adj = adjacency.

exp=explode.

**Supplementary Figures and Tables**

**Table S1.** Exhaustive list of extracted data from included studies

| **Category** | **Data points** |
| --- | --- |
| Study characteristics | First author, country, study interval, study design |
| Patient selection | Inclusion and exclusion criteria |
| Operative details | Operation type and indication |
| Sarcopenia assessment | Definition, consensus reference(s), parameter(s), software/device used (as applicable), cut-off value |
| Patient characteristics | Sample size, age at time of surgery, sex, BMI |
| Postoperative morbidity and mortality | Morbidity (overall^a^, major^b^ and moderate-to-minor^c^ complications according to the CD classification scale), short-term mortality (in-hospital, 30-/90-day follow-up), hospital LOS (postoperative and total), unplanned hospital readmissions and reoperations, and long-term mortality (6-months, 1-/2-years) |

BMI, body mass index; CD, Clavien-Dindo.

^a^ Defined as the occurrence of at least one CD grade I-V postoperative complication.

^b^ At least one CD grade III-V postoperative complication.

^c^ At least one CD grade I-II postoperative complication.

**Table S2**. Quality assessment of included studies using the QUality In Prognosis Studies (QUIPS) tool

| **Study** | **Bias domain** | | | | | |
| --- | --- | --- | --- | --- | --- | --- |
|  | **Study participation** | **Study attrition** | **Prognostic factor measurement** | **Outcome measurement** | **Study confounding** | **Statistical analysis and reporting** |
| Aoki ’22 [31] | Low | Low | Low | Low | Low | Low |
| Aycicek ’12 [32] | High | Low | Low | Moderate | High | Low |
| Berardi ’20 [33] | Low | Low | Low | Low | Low | Low |
| Chen ’16 [34] | Low | Low | Low | Low | Low | Low |
| Chen ’17 [35] | Low | Low | Low | Low | Low | Low |
| Chen ’19 [36] | Low | Low | Low | Low | Low | Low |
| Erkul ’22 [37] | Low | Low | Low | Low | Low | Low |
| Fukuda ’16 [38] | Moderate | Low | Low | Moderate | Low | Low |
| Harimoto ’17 [39] | Low | Low | Low | Low | Low | Low |
| Kaido ’17 [40] | Low | Low | Low | Low | Moderate | Moderate |
| Kurita ’20 [41] | Low | Low | Low | Low | Low | Low |
| Lou ’19 [42] | Low | Low | Low | Low | Low | Low |
| Makiura ’18 [43] | Low | Low | Low | Moderate | Low | Low |
| Matsui ’21 [44] | Low | Low | Low | Low | Low | Low |
| Sato ’16 [45] | Low | Low | Low | Low | Low | Low |
| Sehouli ’21 [46] | Low | Low | Low | Low | Low | Low |
| Welch ’19 [47] | Moderate | High | Low | Low | Moderate | Moderate |
| Zhuang ’20 [48] | Low | Low | Low | Low | Low | Low |
| Zhang ’22 [49] | Low | Low | Low | Low | Low | Low |
| Zhuang ’22 [50] | Low | Low | Low | Low | Low | Low |

**Table S3.** Patient demographic characteristics in each included study

| **Study** | **Total sample size (S, NS)** | **Age in years (S, NS), mean±SD** | **Sex (S, NS), n*** | **BMI in kg/m^2^ (S, NS), mean±SD** |
| --- | --- | --- | --- | --- |
| Aoki ’22 [31] | 180 (19, 161) | 77.9±4.0, 71.0±7.5 | 15/4, 87/74 | 20.1±1.4, 22.1±3.5 |
| Aycicek ’12 [32] | 49 (14, 35) | 71.2±4.6, 71.4±5.7 | 1/13, 24/11 | 23.3±5.0, 26.7±4.8 |
| Berardi ’20 [33] | 234 (68, 166) | 65.7±15.5, 66.9±10.7 | 31/37, 127/39 | 26.6±5.9, 27.0±6.5 |
| Chen ’16 [34] | 158 (39, 119) | 72.9±6.5, 64.9±8.5 | 31/8, 95/24 | 20.5±2.5, 22.8±2.7 |
| Chen ’17 [35] | 376 (92, 284) | 71.0±11.6, 62.2±11.8 | 44/48, 184/100 | 21.5±3.0, 23.5±3.0 |
| Chen ’19 [36] | 313 (37, 276) | 74.0±10.4, 61.0±12.0 | 23/14, 206/70 | 20.3±2.6, 23.2±3.0 |
| Erkul ’22 [37] | 146 (31, 115) | 72.0±8.7, 61.6±11.3 | 26/5, 77/38 | 22.9±4.6, 26.4±5.1 |
| Fukuda ’16 [38] | 99 (21, 78) | 77.4±4.8, 75.5±5.2 | 19/2, 47/31 | 19.8±3.0, 21.9±3.8 |
| Harimoto ’17 [39] | 102 (24, 78) | 55.4±11.7, 55.9±9.4 | 7/17, 38/39 | 22.2±3.2, 24.3±3.4 |
| Kaido ’17 [40] | 72 (10, 62) |  |  |  |
| Kurita ‘20^#^ [41] | 161 (19, 142) |  |  |  |
| Lou ’19 [42] | 206 (14, 192) | 74.8±5.1, 63.3±9.9 | 9/5, 152/40 | 24.2±1.0, 25.2±1.9 |
| Makiura ’18 [43] | 98 (31, 67) | 69.7±7.8, 65.4±7.6 | 24/7, 59/8 | 19.5±2.7, 21.8±2.8 |
| Matsui ‘21^#^ [44] | 96 (35, 61) | 71.4±11.9, 66.3±9.9 | 31/4, 31/30 | 21.9±3.8, 22.9±3.5 |
| Sato ‘16^#^ [45] | 293 (54, 239) |  |  |  |
| Sehouli ’21 [46] | 226 (68, 158) |  |  |  |
| Welch ’19 [47] | 7 (2, 5) |  |  |  |
| Zhuang ’20 [48] | 883 (167, 716) | 73.5±7.5 | 121/46 | 21.5±3.0 |
| Zhang ’22 [49] | 507 (73, 434) | 73.5±6.4, 62.6±9.7 | 53/20, 314/120 | 22.1±3.0, 23.0±2.8 |
| Zhuang ’22 [50] | 1,215 (241, 974) | 74.0±8.1 | 176/65, 710/264 | 21.4±2.9, 22.9±3.1 |

n, number of patients; S, sarcopenic; sd, standard deviation.

*Patient sex reported as male/female.

#Sarcopenia defined by clinical measures (i.e., low handgrip strength) only.

**Figure S1.** Forest plot of postoperative morbidity following elective abdominal surgery in *(A)* the overall cohort (incidence), sarcopenic (S) *versus* non-sarcopenic (NS) patients on *(B)* meta-analysis and *(C)* after adjusting for confounding patient factors using multivariate regression analysis results from individual studies, and *(D)* major as well as *(E)* moderate-to-minor complications.


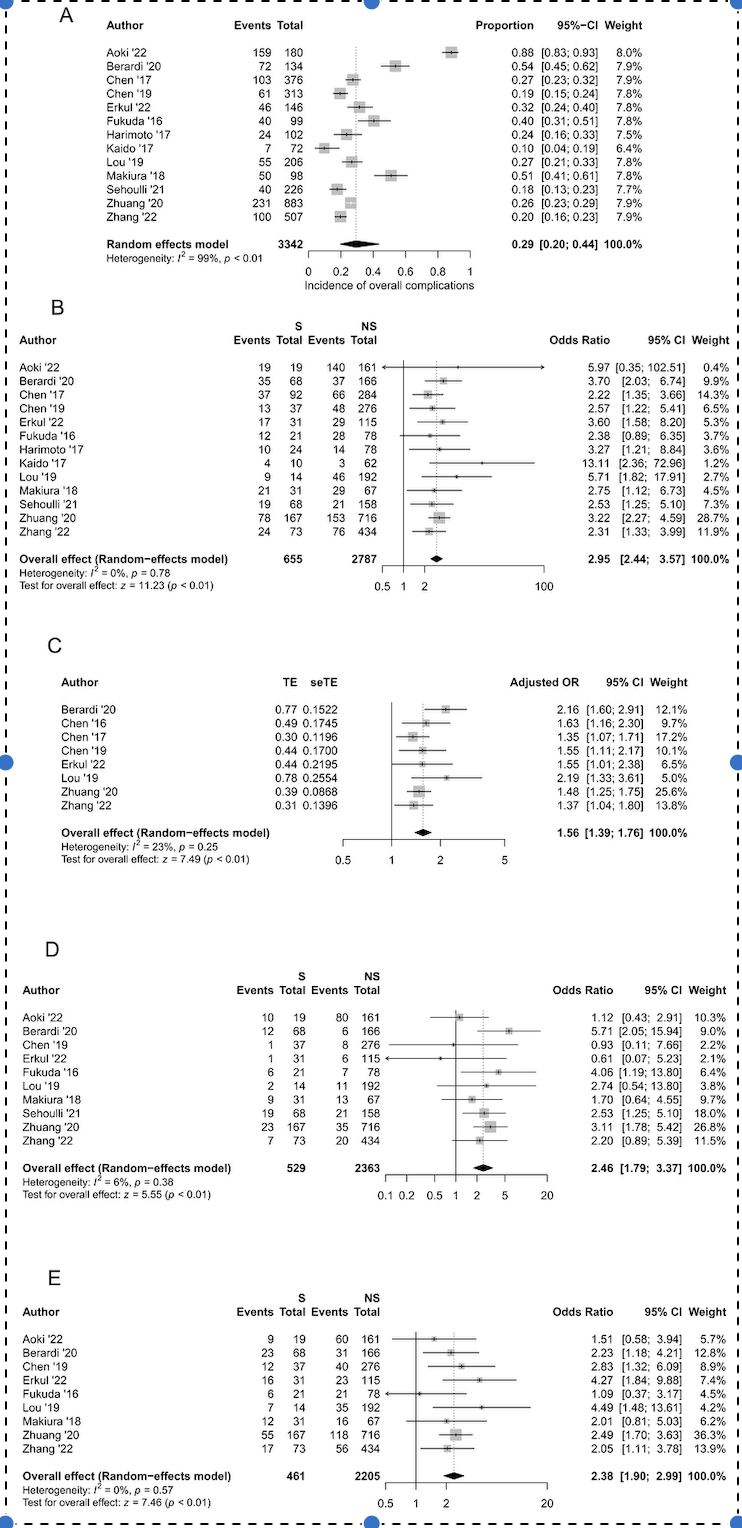


**Figure S2.** Forest plot of mortality after elective abdominal surgery in *(A)* the overall cohort (incidence) and sarcopenic *versus* non-sarcopenic patients during the *(B)* index hospital stay (in-hospital mortality) and in the *(C)* 30-day and *(D)* 90-day follow-up periods.


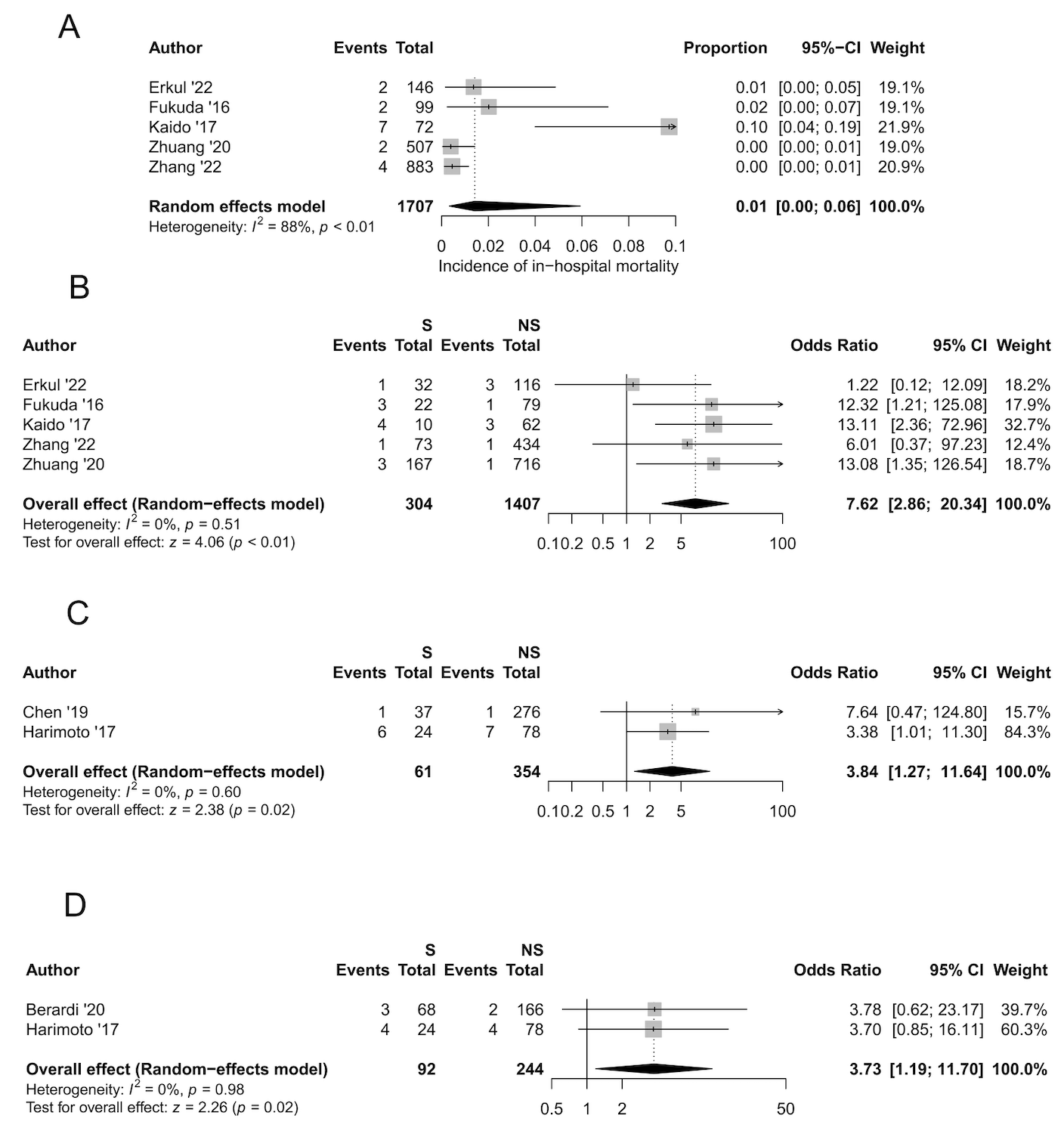


**Table S4.** Subgroup meta-analyses in sarcopenic *versus* non-sarcopenic patients after gastric surgery

| **Outcome** | | **No. of studies** | **Reference(s)** | **Effect size** ^a^ | **95% C.I** | **I^2^, %** |
| --- | --- | --- | --- | --- | --- | --- |
| **PRIMARY ENDPOINTS** | Postoperative complications ^b,c^ |  |  |  |  |  |
|  | Overall (CD grade I-V) | 6 | 36-38,42,48,50 | 2.99 | **2.34 – 3.83** | 0 |
|  | Major (CD grade III-V) | 6 | 36-38,42,48,50 | 2.66 | **1.77 – 4.01** | 0 |
|  | Minor (CD grade I-II) | 6 | 36-38,42,48,50 | 2.53 | **1.94 – 3.29** | 8 |
|  | Postoperative mortality (short-term) |  |  |  |  |  |
|  | In-hospital | 4 | 37,38,48,50 | 5.86 | **1.77 – 19.37** | 0 |
|  | 30-day | 1 | 36 | 7.64 | 0.47 – 124.80 | - |
|  | 90-day |  |  |  |  |  |
| **SECONDARY ENDPOINTS** | Unplanned hospital readmissions ^c,d^ | 4 | 36,37,42,50 | 1.80 | 0.81-4.00 | 40 |
|  | Unplanned reoperations ^c^ | 2 | 36,37 | 1.86 | 0.36 – 9.59 | 0 |
|  | Length of hospital stay (days) ^e^ |  |  |  |  |  |
|  | Postoperative | 5 | 36-38,42,50 | 2.05 | **1.26-2.83** | 0 |
|  | Total |  |  |  |  |  |
|  | Postoperative mortality (long-term) ^d^ |  |  |  |  |  |
|  | 6-month |  |  |  |  |  |
|  | 1-year | 1 | 50 | 4.77 | **1.60 – 14.17** | - |
|  | 2-year |  |  |  |  |  |

CI, confidence interval.

Blank rows relate to endpoints that were not reported in patients undergoing gastric surgery.

* 95% C.I that did not cross the no effect line (0 for continuous endpoints and 1 for categorical endpoints) were considered to indicate a statistically significant difference and are **bolded.**

^a^ An OR >1 and MD >0 favor sarcopenic patients, whereas OR <1 and MD <0 favor non-sarcopenic patients.

^b^ Defined according to the Clavien-Dindo classification system for postoperative complications.

^c^ Effect size presented as the odds ratio (OR).

^d^ Within 30-days of hospital discharge (unless otherwise stated).

^e^ Effect size presented as the mean difference (MD).

**Table S5.** Subgroup meta-analyses in sarcopenic *versus* non-sarcopenic patients after oesophageal surgery

| **Outcome** | | **No. of studies** | **Reference(s)** | **Effect size** ^a^ | **95% C.I** | **I^2^, %** |
| --- | --- | --- | --- | --- | --- | --- |
| **PRIMARY ENDPOINTS** | Postoperative complications ^b,c^ |  |  |  |  |  |
|  | Overall (CD grade I-V) | 1 | 43 | 2.75 | **1.12-6.73** | - |
|  | Major (CD grade III-V) | 1 | 43 | 1.70 | 0.64 – 4.55 | - |
|  | Minor (CD grade I-II) | 1 | 43 | 2.01 | 0.81 – 5.03 | - |
|  | Postoperative mortality (short-term) ^c^ |  |  |  |  |  |
|  | In-hospital |  |  |  |  |  |
|  | 30-day |  |  |  |  |  |
|  | 90-day |  |  |  |  |  |
| **SECONDARY ENDPOINTS** | Unplanned hospital readmissions ^c,d^ | 2 | 41,43 | 2.96 | **1.22 – 7.22** | 0 |
|  | Unplanned reoperations ^c^ |  |  |  |  |  |
|  | Length of hospital stay (days) ^e^ |  |  |  |  |  |
|  | Postoperative | 2 | 41,43 | 12.91 | -5.61 – 31.42 | 87 |
|  | Total |  |  |  |  |  |
|  | Postoperative mortality (long-term) ^d^ |  |  |  |  |  |
|  | 6-month |  |  |  |  |  |
|  | 1-year |  |  |  |  |  |
|  | 2-year | 1 | 43 | 2.18 | 0.92 – 5.17 | - |

CI, confidence interval.

Blank rows relate to endpoints that were not reported in patients undergoing gastric surgery.

* 95% C.I that did not cross the no effect line (0 for continuous endpoints and 1 for categorical endpoints) were considered to indicate a statistically significant difference and are **bolded.**

^a^ An OR >1 and MD >0 favor sarcopenic patients, whereas OR <1 and MD <0 favor non-sarcopenic patients.

^b^ Defined according to the Clavien-Dindo classification system for postoperative complications.

^c^ Effect size presented as the odds ratio (OR).

^d^ Within 30-days of hospital discharge (unless otherwise stated).

^e^ Effect size presented as the mean difference (MD).

**Table S6.** Subgroup meta-analyses in sarcopenic *versus* non-sarcopenic patients after colorectal surgery

| **Outcome** | | **No. of studies** | **Reference(s)** | **Effect size** ^a^ | **95% C.I** | **I^2^, %** |
| --- | --- | --- | --- | --- | --- | --- |
| **PRIMARY ENDPOINTS** | Postoperative complications ^b,c^ |  |  |  |  |  |
|  | Overall (CD grade I-V) | 1 | 35 | 2.22 | **1.35 – 3.66** | - |
|  | Major (CD grade III-V) |  |  |  |  |  |
|  | Minor (CD grade I-II) |  |  |  |  |  |
|  | Postoperative mortality (short-term) |  |  |  |  |  |
|  | In-hospital |  |  |  |  |  |
|  | 30-day |  |  |  |  |  |
|  | 90-day |  |  |  |  |  |
| **SECONDARY ENDPOINTS** | Unplanned hospital readmissions ^c,d^ | 1 | 35 | 2.08 | 0.34 – 12.65 | - |
|  | Unplanned reoperations ^c^ |  |  |  |  |  |
|  | Length of hospital stay (days) ^e^ |  |  |  |  |  |
|  | Postoperative |  |  |  |  |  |
|  | Total | 1 | 35 | 1.40 | -0.25 – 3.05 | - |
|  | Postoperative mortality (long-term) ^d^ |  |  |  |  |  |
|  | 6-month |  |  |  |  |  |
|  | 1-year |  |  |  |  |  |
|  | 2-year |  |  |  |  |  |

CI, confidence interval.

Blank rows relate to endpoints that were not reported in patients undergoing gastric surgery.

* 95% C.I that did not cross the no effect line (0 for continuous endpoints and 1 for categorical endpoints) were considered to indicate a statistically significant difference and are **bolded.**

^a^ An OR >1 and MD >0 favor sarcopenic patients, whereas OR <1 and MD <0 favor non-sarcopenic patients.

^b^ Defined according to the Clavien-Dindo classification system for postoperative complications.

^c^ Effect size presented as the odds ratio (OR).

^d^ Within 30-days of hospital discharge (unless otherwise stated).

^e^ Effect size presented as the mean difference (MD).

**Table S7.** Subgroup meta-analyses in sarcopenic *versus* non-sarcopenic patients following liver transplantation

| **Outcome** | | **No. of studies** | **Reference(s)** | **Effect size** ^a^ | **95% C.I** | **I^2^, %** |
| --- | --- | --- | --- | --- | --- | --- |
| **PRIMARY ENDPOINTS** | Postoperative complications ^b,c^ |  |  |  |  |  |
|  | Overall (CD grade I-V) | 3 | 33,39,40 | 4.00 | **2.40 – 6.66** | 3 |
|  | Major (CD grade III-V) | 1 | 33 | 5.71 | **2.05 – 15.94** | - |
|  | Minor (CD grade I-II) | 1 | 33 | 2.23 | **1.18 – 4.21** | - |
|  | Postoperative mortality (short-term) |  |  |  |  |  |
|  | In-hospital | 1 | 40 | 13.11 | **2.36 – 72.96** | - |
|  | 30-day | 1 | 39 | 3.38 | **1.01 – 11.30** | - |
|  | 90-day | 2 | 33,39 | 3.73 | **1.19 – 11.70** | 0 |
| **SECONDARY ENDPOINTS** | Unplanned hospital readmissions ^c,d^ | 1 | 35 | 2.08 | 0.34 – 12.65 | - |
|  | Unplanned reoperations ^c^ |  |  |  |  |  |
|  | Length of hospital stay (days) ^e^ |  |  |  |  |  |
|  | Postoperative | 1 | 39 | 13.00 | **1.05 – 24.95** | - |
|  | Total | 1 | 33 | 4.00 | **2.51 – 5.49** | - |
|  | Postoperative mortality (long-term) ^d^ |  |  |  |  |  |
|  | 6-month | 1 | 39 | 4.00 | **1.15 – 13.88** | - |
|  | 1-year |  |  |  |  |  |
|  | 2-year |  |  |  |  |  |

CI, confidence interval.

Blank rows relate to endpoints that were not reported in patients undergoing gastric surgery.

* 95% C.I that did not cross the no effect line (0 for continuous endpoints and 1 for categorical endpoints) were considered to indicate a statistically significant difference and are **bolded.**

^a^ An OR >1 and MD >0 favor sarcopenic patients, whereas OR <1 and MD <0 favor non-sarcopenic patients.

^b^ Defined according to the Clavien-Dindo classification system for postoperative complications.

^c^ Effect size presented as the odds ratio (OR).

^d^ Within 30-days of hospital discharge (unless otherwise stated).

^e^ Effect size presented as the mean difference (MD).

# **Table S8.** Covariates included within the multivariate regression analysis model for each study.

| **Author** | **Outcome** | **Multivariate regression covariates** |
| --- | --- | --- |
| Aoki ’22 [31] | Overall survival | Sarcopenia ((SMI <7.0 kg/m^2^ [men] or <6.0 kg/m^2^ [women]) AND (HGS <27 kg [men] or <16 kg [women])), preoperative CA19-9 (≥114 *vs.* <114 U/mL), combined vascular resection, intraoperative blood loss (≥1,205 *vs.* <1,205g), microscopic lymphatic invasion, lymph node metastases, adjuvant chemotherapy completion |
| Aycicek ’12 [32] |  |  |
| Berardi ’20 [33] | Overall morbidity^a^ | ASA score, sarcopenia ((SMI <53.5 cm^2^/m^2^ [men] or <40.8 cm^2^/m^2^ [women]) AND (HGS <30 kg [men] or <20 kg [women])), liver cirrhosis, preoperative portal hypertension, open surgery, major hepatectomy, biliary reconstruction, intraoperative blood loss, operative time |
| Chen ’16 [34] | Overall morbidity^a^ | Age (≥75 *vs.* <75 years), ASA score (≥III *vs.* I-II), CCI score (≥2 *vs.* <2), NRS 2002 score (≥3 *vs.* <3), sarcopenia (((SMI <40.8 cm^2^/m^2^ [men] or <34.9 cm^2^/m^2^ [women]) AND (HGS <26 kg [men] or <18 kg [women])) AND/OR (GS <0.8 m/s)), tumor location at cardia, laparoscopic surgery, combined organ resection |
| Chen ’17 [35] | Overall morbidity^a^ | Age (≥65 *vs.* <65 years), laparoscopic-assisted surgery, visceral obesity (VFA >130 cm^2^ [men] OR >90 cm^2^ [women]), sarcopenia (((SMI <40.8 cm^2^/m^2^ [men] or <34.9 cm^2^/m^2^ [women]) AND (HGS <26 kg [men] or <18 kg [women])) AND/OR (GS <0.8 m/s)) |
| Chen ’19 [36] | Overall morbidity^a^ | Age (≥65 *vs.* <65 years), sarcopenia (((SMI <40.8 cm^2^/m^2^ [men] or <34.9 cm^2^/m^2^ [women]) AND (HGS <26 kg [men] or <18 kg [women])) AND/OR (GS <0.8 m/s)), CCI score (≥1 *vs.* 0), hypoalbuminemia (<35 *vs.* ≥35 g/L), anemia (<120 *vs.* ≥120 g/L [men] OR <110 *vs.* ≥110 g/L [women]) |
| Erkul ’22 [37] | Overall morbidity^a^ | Age, sex (male *vs.* female), albumin level, BMI, splenectomy, sarcopenia (((SMI <43 cm^2^/m^2^ [men] with BMI <25 kg/m^2^ or <53 cm^2^/m^2^ with BMI ≥25 kg/m^2^ or <41 cm^2^/m^2^ [women]) AND ((HGS <27 kg [men] or <16 kg [women]) AND/OR (GS <0.8 m/s)) |
| Fukuda ’16 [38] | Major complications^b^ | Age (≥75 *vs.* <75 years), sex (male *vs.* female), hypoalbuminemia (<4 *vs.* ≥4 g/dL), ASA score (≥III *vs.* I-II), neoadjuvant chemotherapy, type of surgery (total *vs.* partial gastrectomy), lymph node dissection (D2 or higher *vs.* D0 or D1), intraoperative blood loss (≥400 *vs.* <400 mL), operative time (≥240 *vs.* <240 mins), UICC pathological stage (III or IV *vs.* I or II), sarcopenia (((SMI ≤8.87 kg/m^2^ [men] or ≤6.42 kg/m^2^ [women]) AND ((HGS <30 kg [men] or < 20 kg [women])) AND/OR (GS ≤0.8 m/s)) |
| Harimoto ’17 [39] | 6-month mortality | Recipient age, donor age, sex (male *vs.* female), recipient status (hospitalized *vs.* home), BMI (≥30 *vs.* <30 kg/m^2^), diabetes mellitus, MELD score (≥20 *vs.* <20), hepatocellular carcinoma, major vessel shunt, GV/SLV ratio (<35 *vs.* ≥35), portal vein pressure at laparotomy, sarcopenia ((SMA <75% of calculated) AND (HGS <26 kg [men] or <18 kg [women])) |
| Kaido ’17 [40] |  |  |
| Kurita ’20 [41] | Postoperative pneumonia | Age, FEV1.0%, postoperative recurrent laryngeal nerve palsy, sarcopenia ((SMI <47.1 cm^2^/m^2^) AND (HGS <27 kg [men] or <16 kg [women])) |
| Lou ’19 [42] | Overall morbidity^a^ | Age (≥65 *vs.* <65 years), sarcopenia (((SMI <40.8 cm^2^/m^2^) AND ((HGS <26 kg [men] or <18 kg [women])) AND/OR (GS <0.8 m/s)) |
| Makiura ’18 [43] | 90-day unplanned hospital readmission | Age (≥70 *vs.* <70 years), sex (male *vs.* female), tumor stage (≥3 *vs.* <3), FILS (≤6 *vs.* >6), postoperative complications, sarcopenia (((SMI <7.0 kg/m^2^ [men] or <5.7 kg/m^2^ [women]) AND (HGS <26 kg [men] or <18 kg [women])) AND/OR (GS <0.8 m/s)) |
|  | Overall survival | Age (≥70 *vs.* <70 years), sex (male *vs.* female), tumor stage (≥3 *vs.* <3), sarcopenia (((SMI <7.0 kg/m^2^ [men] or <5.7 kg/m^2^ [women]) AND (HGS <26 kg [men] or <18 kg [women])) AND/OR (GS <0.8 m/s)) |
| Matsui ’21 [44] | Overall survival | Age (≥70 *vs.* <70 years), surgical approach (open *vs.* laparoscopic), pathological stage (≥III *vs.* <III), sarcopenia ((SMI <42.66 cm^2^/m^2^ [men] or <34.99 cm^2^/m^2^ [women]) AND (HGS <34.7 kg [men] or <14.0 kg [women])) |
| Sato ’16 [45] | Overall morbidity^a^ | Sex (male *vs.* female), surgical procedure (total *vs.* subtotal gastrectomy), splenectomy±pancreatectomy, sarcopenia (HGS ≤15.8 kg [men] or ≤13.8 kg [women]) |
| Sehouli ’21 [46] | Major complications^b^ |  |
|  | Overall survival | Age (≥65 *vs.* <65 years), major 30-day postoperative complications (CD ≥III *vs.* <III), ECOG performance status (>1 *vs.* ≤1), FIGO stage (≥IIIa *vs.* <IIIa), hypoalbuminaemia (<35.5 *vs.* ≥35 g/L), phase angle alpha (<4.5 *vs.* ≥4.5 degrees), NRS (≥3 *vs.* <3), BMI (≥25 kg/m^2^ *vs.* <25 kg/m^2^), weight loss (≥10% *vs.* <10% last 3 months), extracellular mass to body cell mass index (>1.3 *vs.* ≤1.3), fat mass (>39 kg *vs.* ≤39 kg), sarcopenia (SMI ≤27% *vs.* >27%) |
| Welch ’19 [47] |  |  |
| Zhuang ’20 [48] | Overall morbidity^a^ | Age (≥75 *vs.* <75 years), sarcopenia (((SMI <40.8 cm^2^/m^2^ [men] or <34.9 cm^2^/m^2^ [women]) AND (HGS <26 kg [men] or <18 kg [women])) AND/OR (GS ≤0.8 m/s)), CCI (1 *vs.* 0 and ≥2 *vs.* 0), ASA score (≥III *vs.* II or ≥III *vs.* I), hypoproteinemia, laparoscopic surgery |
|  | Overall survival | Age (≥75 *vs.* <75 years), BMI (≥25 kg/m^2^ *vs.* 18.5-25 kg/m^2^), NRS 2002 score (≥3 *vs.* <3), sarcopenia (((SMI <40.8 cm^2^/m^2^ [men] or <34.9 cm^2^/m^2^ [women]) AND (HGS <26 kg [men] or <18 kg [women])) AND/OR (GS ≤0.8 m/s)), hypoproteinemia, histology type (undifferentiated *vs.* differentiated, TNM stage (II *vs.* I or III *vs.* I), type of resection (total *vs.* subtotal), combined resection, laparoscopic surgery, postoperative complications (CD II *vs.* 0 or CD III-V *vs.* 0) |
| Zhang ’22 [49] | Overall morbidity^a^ | Age (≥65 *vs.* <65 years), sex (male *vs.* female), BMI, anemia (<120 *vs.* ≥120 g/L [men] or <110 *vs.* ≥110 g/L [female]), hypoalbuminaemia (<35 *vs.* ≥35 g/L), ASA score (≥III *vs.* I-II), CCI (≥1 *vs.* 0), history of abdominal surgery, laparoscopic-assisted surgery, type of resection, type of reconstruction, operative time, intraoperative blood loss, intraoperative blood transfusions, sarcopenia ((SMI <40.8 cm^2^/m^2^ [men] or <34.9 cm^2^/m^2^ [women]) AND (HGS <26 kg [men] or <18 kg [women])) |
|  | Overall survival |  |
| Zhuang ’22 [50] | Overall survival | Age (≥75 *vs.* <75 years), sex (male *vs.* female), BMI (≤18.5 kg/m^2^ *vs.* 18.5-25 kg/m^2^), NRS 2002 score (≥3 *vs.* <3), sarcopenia ((SMI <40.8 cm^2^/m^2^ [men] or <34.9 cm^2^/m^2^ [women]) AND (HGS <26 kg [men] or <18 kg [women])), cachexia, hypoproteinemia, tumor size (>50 *vs.* ≤50 mm), histologic type (undifferentiated *vs.* differentiated), TNM stage (II or III *vs.* I), type of resection (total *vs.* subtotal), combined resection, surgical approach (laparoscopic *vs.* open), adjuvant chemotherapy, postoperative complications^a^ |

ASA, American Society of Anesthesiologists; BMI, body mass index; CCI, Charlson Comorbidity Index; CD, Clavien-Dindo; ECOG, Eastern Cooperative Oncology Group; FEV1.0, forced expiratory volume in 1 second; FIGO, Federation International of Gynecology and Obstetrics; FILS, food intake level scale; GS, gait speed; GV, graft volume; HGS, hand-grip strength; MELD, Model for End-stage Liver Disease; NRS, Nutritional Risk Screening; SLV, standard liver volume; SMA, skeletal muscle area; SMI, skeletal muscle index; UICC, Union for International Cancer Control; VFA, visceral fat area.

^a^ Defined as the occurrence of at least one CD grade I (or higher) complication following elective abdominal surgery.

^b^ Occurrence of at least one CD grade III (or higher) postoperative complication.

Empty cells are used to indicate studies where multivariate regression analysis was not performed.
